# Epinephrine’s effects on cerebrovascular and systemic hemodynamics during cardiopulmonary resuscitation: metabolic changes may limit the persistence of the effect

**DOI:** 10.1186/s13054-020-03378-4

**Published:** 2021-02-16

**Authors:** Romain Jouffroy, Benoît Vivien

**Affiliations:** 1 Service de Médecine Intensive Réanimation, Hôpital Universitaire Ambroise Paré, APHP. Université Paris Saclay, Assistance Publique - Hôpitaux de Paris, Boulogne Billancourt, France; 2 Université de Versailles Saint Quentin, Versailles, France; 3 SAMU de Paris, Service d’Anesthésie-Réanimation, Hôpital Universitaire Necker - Enfants Malades, APHP. Centre, Assistance Publique - Hôpitaux de Paris, Paris, France; 4 Université de Paris, Paris, France

To the Editor,

Mavroudis et al. [1] recently reported that, in a swine model of pediatric in-hospital cardiac arrest, epinephrine increases cerebral blood flow (CBF) and cerebral tissue oxygenation, its effects waning after the third epinephrine dose. The authors should be congratulated for this study concerning one of the main medications used for cardiopulmonary resuscitation (CPR) whose safety and efficacy remain under debate [2, 3]. Nevertheless, we believed that some points of their study should be pointed out. First, it seems surprising to use epinephrine as a first-line treatment for a shockable cardiac arrest, for which defibrillation is the recommended first-line treatment [2]. Second, the animal model used, i.e., a swine model of asphyxia associated cardiac arrest, resulting in acidosis and hypoxemia, may partly explain the lack of epinephrine efficacy on CBF and cerebral tissue oxygenation observed after the third dose (see additional File 2) because of the negative effects of hypoxemia and acidosis to the response to sympathomimetic agents [4]. Acidosis and hypoxemia impair the vascular alpha-1-sympathomimetic receptor response and limit the epinephrine efficacy on blood pressure and coronary perfusion increases [4]. Mavroudis et al. [1] results suggest that the previously reported deleterious effects of cumulative epinephrine doses [2, 3] are probably not related to epinephrine itself but to its lack of efficacy due to the underlying metabolic alterations.

In conclusion, we fully agree with Mavroudis et al. [1], that, despite the exact mechanisms of epinephrine’s effects on CBF and cerebral oxygenation, CPR methods, including epinephrine administration, aim to maintain CBF in order to limit cerebral hypoperfusion and neurologic injury. Moreover, even if CPR methods allow to maintain CBF, pending cardiac arrest etiological treatment, short and long-term survival increase requires a true bundle of care, including, CPR methods and cerebral protection, implemented complementarily to the chain of survival [5].
