# Supplementary material for: Epinephrine’s effects on cerebrovascular and systemic hemodynamics during cardiopulmonary resuscitation: metabolic changes may limit the persistence of the effect
Source: Crit Care. 2021 Feb 16;25:67. doi: 10.1186/s13054-020-03378-4 (PMC7887812; doi:10.1186/s13054-020-03378-4)
Supplement: Related texts

# Reply: Authors’ response

**Authors:** Constantine D. Mavroudis, Ryan W. Morgan, Morgan A. Berg, Todd J. Kilbaugh

We appreciate the insightful remarks of Drs. Jouffroy and Vivien regarding our laboratory study of the cerebral hemodynamic response to epinephrine during cardiopulmonary resuscitation [1]. We wish to respond to the authors’ commentary regarding the impact of the cardiac arrest model utilized on the study’s findings.

Our study utilized an asphyxia-associated model of cardiac arrest because a substantial proportion of in-hospital cardiac arrests in children and adults occur in the setting of respiratory failure. After seven minutes of asphyxia, ventricular fibrillation (VF) was electrically induced. This was done principally for the purpose of ensuring a consistent minimum duration of cardiac arrest in which to study intra-arrest physiology (i.e., animals do not regain spontaneous circulation until defibrillation is provided at a set timepoint). In some laboratory models, CPR is provided to animals with asphyxia-associated pulseless electrical activity (PEA) or asystole. This offers clinical relevance but is frequently followed by prompt ROSC, which precludes extensive study of intra-arrest physiology. Drs. Jouffroy and Vivien are correct in their assertion that timely defibrillation is of paramount importance during a VF arrest and should not be delayed in order to administer epinephrine. However, in this established model of cardiac arrest, allowing VF to continue untreated for ten minutes prevented changes in underlying rhythm and spontaneous circulation, thus reducing confounding in our analysis of the effects of epinephrine on cerebral hemodynamics.

We agree with the hypothesis that metabolic derangements during cardiac arrest likely play an important role in the observation that epinephrine’s cerebral hemodynamic effects diminish later in the course of CPR. The effects of acidosis on the efficacy of vasopressors and inotropes, especially at the extremes of cardiovascular physiology, is an area of ongoing research. In a 2013 study by Vidal et al, the vasoactive effects of epinephrine and norepinephrine were not negatively affected after exposing ex vivo human mammary artery to moderate (pH 7.2) and even severe (pH 6.8–7.0) acidosis [6]. The deleterious cardiac effects of severe acidosis are well known, and the potential for superimposed acquired pulmonary hypertension and right heart failure during prolonged periods of acidosis and cardiopulmonary resuscitation may certainly confound any potential benefit that epinephrine could have during prolonged resuscitation. The further effects that the myriad physiologic perturbations associated with cardiac arrest and subsequent resuscitation might have on the incompletely developed (and poorly understood) cerebral autoregulatory mechanisms in juvenile animals and humans are an important additional concern, and require further research.

It is important to note that while the asphyxial model studied does in fact lead to acidosis and severe hypoxemia, our previous work with this model demonstrated that after six minutes of CPR, hypoxemia was resolved, PaCO2 was nearly normalized, and pH was higher than at CPR onset [7]. Thus, we are not convinced that epinephrine’s ineffectiveness later in CPR was a function of our asphyxial model, but rather that it may be indicative of metabolic and physiologic changes that occur with prolonged CPR in general. Further dedicated study to elucidate these mechanisms may allow us to target them and optimize cerebral hemodynamics even during prolonged resuscitation.
